# Analytical performance of the selective multianalyser Olympus AU 5200

**DOI:** 10.1155/S1463924693000094

**Published:** 1993

**Authors:** H. Mayer, C. Luley, M. Behnke, H. Wieland

**Affiliations:** Abteilung für Klinische Chemie Universitätslinik der Albert-Lutwigs- Universität Freiburg Germany

## Abstract

The analytical performance of a selective, automatic multianalyser- the Olympus A U5200 - was tested and assessed for practicability, following ECCLSguidelines. Twenty-two analytes were tested and compared with the Olympus A U5000 analyser. A Hitachi 747 analyser was also included in this survey in order to obtain correlation data for ISE measurements.

The imprecision data, expressed as median CV values, were found to be below 2% in series for 21 parameters, and below 3% for 19 paramaters from day to day. Creatinine measured with the kinetic Jaffe method obtained a median CV value of 4% in series, creatine phosphokinase showed the worst imprecision from day to day with a CV of 9%. Slightly better precision values for the majority ofall tests were found on the Olympus AU5200 than on the AU 5000 analyser.

The recovery of the assigned values in 32 commercial control sera was between 95% and 105% for 14 tests. Five of the remaining tests yielded recoveries with deviations between 5% and 10%, deviations above 10% showed albumine, alanine aminotransferase, aspartate aminotransferase and creatine phosphokinase. The accuracy of most test parameters was slightly better on the AU5200 analyser than on the comparison instrument.

The range of linearity ofthe tested methods covered the range stated by the manufacturers; and no sample carry-over was detected. Most parameters tested yielded close correlation to those on the comparison instrument. Amylase measurements on both analysers correlate well but are not comparable without data correction due to different test methods. In addition, no drift effects were observed over a period of 9 hours.

The ion-selective-electrode unit performed well in terms of throughput, precision and stability over time. The whole system showed good practicability with respect to patient sample and reagent handling, a short training period of technicians, ease of system software, maintenence, a robust barcode reader and a flexible host communication procedure.


Journal of Automatic Chemistry, Vol. 15, No. 2 (March-April 1993), pp. 47-64

### Analytical performance of the selective multianalyser Olympus AU 5200

**H. Mayer, C. Luley, M. Behnke and H. Wieland**

*Abteilung für Klinische Chemie, Universitätsklinik der Albert-Lutwigs-Universität, Freiburg, Freiburg, Germany*

*The analytical performance of a selective, automatic multianalyser - the Olympus AU 5200 - was tested and assessed for practicability, following ECCLS guidelines. Twenty-two analytes were tested and compared with the Olympus AU 5000 analyser. A Hitachi 747 analyser was also included in this survey in order to obtain correlation data for ISE measurements.*

*The imprecision data, expressed as median CV values, were found to be below 2% in series for 21 parameters, and below 3% for 19 paramaters from day to day. Creatinine measured with the kinetic Jaffe method obtained a median CV value of 4% in series, creatine phosphokinase showed the worst imprecision from day to day with a CV of 9%. Slightly better precision values for the majority of all tests were found on the Olympus AU 5200 than on the AU 5000 analyser.*

*The recovery of the assigned values in 32 commercial control sera was between 95% and 105% for 14 tests. Five of the remaining tests yielded recoveries with deviations between 5% and 10%, deviations above 10% showed albumine, alanine aminotransferase, aspartate aminotransferase and creatine phosphokinase. The accuracy of most test parameters was slightly better on the AU 5200 analyser than on the comparison instrument.*

*The range of linearity of the tested methods covered the range stated by the manufacturers; and no sample carry-over was detected. Most parameters tested yielded close correlation to those on the comparison instrument. Amylase measurements on both analysers correlate well but are not comparable without data correction due to different test methods. In addition, no drift effects were observed over a period of 9 hours.*

*The ion-selective-electrode unit performed well in terms of throughput, precision and stability over time. The whole system showed good practicability with respect to patient sample and reagent handling, a short training period of technicians, ease of system software, maintenence, a robust barcode reader and a flexible host communication procedure.*

### Introduction

The Olympus AU 5200 series of selective multianalysers is well suited for medium and large laboratories processing several thousand samples per day. This paper reports on the performance characteristics of the AU 5200 analyser comparing it with the AU 5000 analyser. The evaluation procedure followed ECCLS guidelines [1]. Although only a multicentre evaluation allows a truly representative assessment for an analyser, the evaluation data presented here give a first impression of the quality and performance of the instrument.

### The AU 5200

The AU 5200 is constructed following a modular concept that originated from the AU 5000 instrument series. Any analyser consists of up to four basic identical units equipped with eight or 16 reagent lines. The stated sample throughput of 330 samples (300 samples with ISE unit), which is twice as much as that of an AU 5000 analyser, is achieved by two cuvette rings per unit with 240 cuvettes each. In an analyser equipped with eight reagent lines per module, 60 cuvettes are assigned to each chemistry channel (this compares with 24 cuvettes in a similar model of the series AU5000). A list of the main specifications of the AU 5200 analyser is given in table 1, along with a survey of possible configurations. Evaluation data on the comparison instrument AU 5000 has been published by Luley *et al.* [2].

The analyser configuration which was evaluated for this paper consisted of three units with eight reagent lines per unit for chemistry tests, and an additional ISE unit for electrolyte measurements.

### Materials

### *Quality-control materials*

The commercially available control materials used in the evaluation are specified in table 2.

### *Calibrators and standards*

Multianalyte calibrators obtained from Olympus Optical Co. and Merck Company were used to calibrate the respective substrate methods of both reagent suppliers. Aqueous electrolyte standards obtained from Olympus Optical Co. and Boehringer Mannheim GmbH were used to calibrate flame photometry and ISE measurement on the Olympus AU 5000, AU 5200 and Hitachi 747 analysers respectively. To measure linearity of chemistry procedures analyte-spiked serum materials were obtained from Sigma Chemical Company (Multi-Analyte Lintrol, Multi-Enzyme Lintrol, CK-Lintrol, Lipid-Lintrol) or produced by adding pure analyte to a serum pool (iron and bilirubin standard solutions from Sigma Chemicals).

### *Chemistry reagents*

The reagent kits were supplied by Olympus Optical Co. in Hamburg and Merck Company in Darmstadt, Germany. A list of reagents, reagent manufacturers and methods of chemistry testing is given in table 3.

0142-0453/93 $10.00 © 1993 Taylor & Francis Ltd.

*Table 1. Instrument specification for the AU 5200*

| | |
|---|---|
| *General characteristics* | |
| Type of instrument | Fully selective discrete access multianalyser |
| Analytical methods | End-point<br>End-point with serum blanking<br>Kinetic serum start<br>Kinetic trigger start<br>ISE or flame photometry |
| Nature of specimen | Serum, plasma, urine |
| Operation of the system | With and without host computer support, batch and realtime operation |
| Specimen identification | Barcode labelled primary tubes, barcode format includes codabar, 2 of 5 and code 39 |
| Specimen carrier | Straight racks with 10 positions for patient's specimen (white), control sera (green), calibrators (yellow), water and reagent blanks (blue), emergency samples (red) and rerun samples (orange) |
| Sample throughput | 330 samples per hour maximally (as for 8 reagent lines per unit without ISE) |
| Sample tray capacity | 300 specimens (30 racks) |
| Sampling system | Pipettors in ISE unit (single) and each module (double) with liquid level sensor driven by stepping motors |
| Sampling volumes | 3–15 μl (1 μl increment) |
| Sampling dispensing | Four tests |
| Reagent type | Liquid |
| Reagent storage | Refrigerated storage (4–12°C) |
| Reagent dispensor | Two independent pipettors per method |
| Reagent volumes | 250–500 μl total (R1 + R2) volume in 1 μl increments |
| Reagent bottle capacity | 125 ml–2000 ml |
| Reaction rotor | Turntable with two rings of 240 cuvettes each |
| Reaction cuvettes | 480 quartz glass cuvettes per unit, 6 mm path length |
| Reaction temperature | 37°C |
| Reaction incubation | Dry bath with incubation liquid system |
| Reaction time | 9 min 24 s |
| Calibrators | Up to 5 calibrants per test parameter, linear and nonlinear calibration |
| *Optical characteristics* | |
| Light source | Tungsten-Halogen lamp |
| Photometry system | Multiwavelength diffraction grating |
| Wavelength | Fixed array of 10 diodes: 340 nm, 380 nm, 410 nm, 480 nm, 520 nm, 540 nm, 600 nm, 660 nm, 800 nm |
| Light path | 6·0 mm |
| Photo detector | Silicon photo cell |
| Photometric range | 0·00 to 2·00 absorbance units |
| Sensitivity | 0·0001 absorbance units |
| *ISE characteristics* | |
| Analytical system | Vertical block flowcell with ion selective electrodes |
| Analytical items | Chloride, potassium, natrium |
| Sample throughput | 300 samples per hour |
| Sample type | Serum, urine |
| Sample volume | 28 μl |
| Diluent volume | 665 μl |
| *Data processor* | |
| Main cpu | Intel i8086 with i8087 coprocessor |
| Storage devices | 40 Mb hard disk, 31/2 in floppy disk |
| On line interface | RS232 C serial interface, bidirectional data transfer |
| Operating system | CP/M 86 |
| Operator oriented software | Quality control; correlation correction; linear transformation (Y = AX + B); test result editing: abnormal upper/lower value judgement; user defined calculated tests; user defined inter-item data check; reaction curve monitor; automatic instrument start up capability; repeat run assessment |
| *Environment* | |
| Power consumption | 8·0 kVA (as for AU 5231 with 3 units)<br>(60 A max. by 210V single phase AC) |
| Heat output | 6880 Kcal |
| Water consumption | 30 1/hour per unit deionized water |
| Waste drainage | Separately expelled: chemical and non-hazardous waste |

*Analyser configurations*

| Model | Analyser composition (units × reagent lines) | Assay items (with ISE) | Sample throughput/h (with ISE) |
|---|---|---|---|
| AU 5211 | 1 × 16 | 16 (19) | 165 (150) |
| AU 5221 | 2 × 8 | 16 (19) | 330 (300) |
| AU 5223 | 2 × 16 | 32 (35) | 165 (150) |
| AU 5231 | 3 × 8 | 24 (27) | 330 (300) |
| AU 5241 | 4 × 8 | 32 (35) | 330 (300) |

### Methods

### Chemistry methods

Both analysers were operated at 37 °C, enzyme activities were converted to corresponding activities at 25 °C. All methods employ bichromatic measurements. The chemical basis for each method on both the AU 5200 and the AU 5000 is summarized in table 2.

### Electrolyte measurements

Sodium and potassium concentrations were measured by flame photometry on the AU 5000 analyser and indirect potentiometry on the AU 5200 analyser and Hitachi 747 analyser (Boehringer Mannheim, Germany). Chloride was determined by reaction with mercuric nitrate and thiocyanate (chloride reagent obtained from Greiner BioChemica in Flacht, Germany) on the AU 5000 analyser and by indirect potentiometry on the AU 5200 and Hitachi 747 analyser.

*Table 2. Control sera used for the AU 5200 evaluation*

| Control serum | Lot No. | Manufacturer |
|---|---|---|
| Decision 1 | 1101 | Beckman Instruments GmbH, Germany |
| Decision 2 | 1102 | |
| Decision 3 | 1103 | |
| Duotrol | 5036 | Biomed, Germany |
| NobiCon I | 210 099 | Nobis GmbH, Germany |
| NobiCon II | 220 077 | |
| Lyotrol N | 482870 | BioMerieux GmbH, Germany |
| Lyotrol P | 323760 | |
| Monotrol | 629430 A | |
| Clinitrol | 632780 A | |
| Zymotrol | 715540 A | |
| Qualitrol L | 326 | Merck Darmstadt GmbH, Germany |
| Qualitrol N | 390 | |
| Qualitrol H | 325 | |
| Precinorm U | 166 760 | Boehringer Mannheim GmbH, Germany |
| Precipath U | 168 593 | |
| Moni-Trol I | LTD-218 | Baxter Deutschland GmbH, Germany |
| Moni-Trol II | PTD-119 | |
| Lypocheck 1 | 48901 | BioRad Laboratories GmbH, Germany |
| Lyphocheck 2 | 48902 | |
| Kontrollogen L | 62 3131 | Behring Werke AG, Germany |
| Kontrollogen LP | 62 3218 | |
| Control Serum N | O 2933 | Hoffman-La Roche AG, Germany |
| Control Serum P | 3133 | |
| Testpoint 1 | V05623 | Technicon/Bayer AG, Germany |
| Testpoint 2 | V05602 | |
| Validate N | 743 | Organon Teknika GmbH, Germany |
| Validate A | 586 | |
| Biotrol 33 Plus | 574 | Biotrol Paris, France |
| Biotrol 33 Plus path. | 713 | |
| Accutrol Normal | 6165 | Sigma Chemie GmbH, Germany |
| Accutrol Abnormal | 6166 | |

### Precision study

To determine the within-run imprecision, seven different control materials and two serum pools were analysed in a series of 20 assays. The series were repeated three times and the medians of the three CV values were taken as the final results.

The imprecision between-days was determined on 15 different days using the seven control materials mentioned above.

### Assessment of accuracy

Thirty-two commercial control materials (listed in table 2) were analysed in double assays on three days in both the AU 5200 and AU 5000. The medians of the measured values were compared with the assigned values of the different control materials (if available for the methodology used).

### Linearity study

The linearity of each chemistry method was assessed using serum material with high analyte concentrations and 25 equidistant levels of dilution with physiological saline solution or bovine serum albumin (for cholesterol, triglyceride and bilirubin dilutions). The dilutions were prepared using a robotic dilutor system (Tecan RSP 505, Zinsser Analytik). The measured concentrations were plotted against dilution levels and the resulting graphs were inspected visually for linearity.

### Carry-over experiments

Due to the construction principle that each cuvette and each mixer blade is used for one kind of test only and that the reagent lines are totally separated from one another, carry-over is restricted to sample carry-over caused by insufficient washing of subsequent cuvettes, the probe needle or the mixer blade.

Sample carry-over, due to insufficient cuvette washing, was tested by determining the activity of creatine phosphokinase during two complete cuvette wheel cycles (120 samples). All samples were taken from a serum pool with a low CPK activity of 27 U/l (25 °C), except sample numbers 31 to 45 which contained a high activity of 1500 U/l. Thus, during the second cuvette wheel cycle (sample numbers 61 to 120) samples containing the low CPK activity were measured in cuvettes which had been used for the determination of the high CPK activity in the previous cuvette wheel cycle. If the cuvettes were inadequately washed, elevated CPK activity should be measured in cuvette numbers 91 to 105.

In order to detect sample carry-over caused by the probe needle or mixer blade, groups of five samples with low CPK activity and high CPK activity were alternated

*Table 3. Test procedures and reagents used during evaluation*

| Analyte | Method* | Reagent manufacturer | Analyser |
|---|---|---|---|
| *1. Enzymes* | | | |
| Alanine aminotransferase | IFCC | Olympus | AU 5200 |
| | IFCC | Merck | AU 5000 |
| Aspartate aminotransferase | IFCC | Olympus | AU 5200 |
| | IFCC | Merck | AU 5000 |
| Alkaline phosphatase | DGKC | Olympus | AU 5200 |
| | DGKC | Olympus | AU 5000 |
| Amylase | bl. PNP-G7 | Olympus | AU 5200 |
| | Cl-PNP-G7 | Merck | AU 5000 |
| Creatine phosphokinase | CK-NAC, DGKC | Olympus | AU 5200 |
| | CK-NAC, DGKC | Merck | AU 5000 |
| g-glutamyl transferase | GCNA 'Szasz' | Olympus | AU 5200 |
| | GCNa 'Szasz' | Merck | AU 5000 |
| Lactate dehydrogenase | SCE | Olympus | AU 5200 |
| | SCE | Merck | AU 5000 |
| *2. Substrates* | | | |
| Albumin | Bromcresol green | Olympus | AU 5200 |
| | Bromcresol green | Merck | AU 5000 |
| Bilirubin | DCA | Olympus | AU 5200 |
| | DPD | Merck | AU 5000 |
| Calcium | o-Cresolphthaleine | Olympus | AU 5200 |
| | o-Cresolphthaleine | Olympus | AU 5000 |
| Cholesterol | CHOD-PAP | Olympus | AU 5200 |
| | CHOD-PAP | Olympus | AU 5000 |
| Creatinine | kinetic Jaffe | Olympus | AU 5200 |
| | enzymatic PAP | Merck | AU 5200 |
| | enzymatic PAP | Merck | AU 5000 |
| Glucose | Hexokinase | Olympus | AU 5200 |
| | Glucose dehydrogenase | Merck | AU 5000 |
| Phosphate | Ammoniumheptamolybdate | Olympus | AU 5200 |
| | Ammoniumheptamolybdate | Merck | AU 5000 |
| Iron | Nitroso-PSAP | Olympus | AU 5200 |
| | Ferrozine | Merck | AU 5000 |
| Total protein | Biuret | Olympus | AU 5200 |
| | Biuret | Olympus | AU 5000 |
| Triglycerides | GPO-PAP | Olympus | AU 5200 |
| | GPO-PAP | Olympus | AU 5000 |
| Urea | GLDH | Olympus | AU 5200 |
| | GLDH | Merck | AU 5000 |
| Uric acid | Uricase/PAP | Olympus | AU 5200 |
| | Uricase/PAP | Merck | AU 5000 |
| *3. Electrolytes* | | | |
| Sodium | ISE indirect | | AU 5200 |
| | Flame photometry | | AU 5000 |
| | ISE indirect | | Hitachi 747 |
| Potassium | ISE indirect | | AU 5200 |
| | Flame photometry | | AU 5000 |
| | ISE indirect | | Hitachi 747 |
| Chloride | ISE indirect | | AU 5200 |
| | Hg-Thiocyanate | Greiner | AU 5000 |
| | ISE indirect | | Hitachi 747 |

* IFCC = International Federation of Clincial Chemistry; DGKC = Deutsche Gesellschaft für Klinische Chemie; PNP-G7 = p-Nitrophenyl maltoheptaoside; CK-NAC = Creatine kinase, N-acetylcysteine activated; GCNA 'Szasz' = L-Glutamyl-3-carboxy-4-nitroanilide: G. Szasz u. M, Klin. Chem. u. Klin. Biochem. 12,228 (1974); SCE = Scandinavian Committee on Enzymes; DCA = 2,4-Dichloraniline-diazonium salt; DPD = 2,5-Dichlorophenyl-diazonium salt; Nitroso-PSAP = 2-Nitroso-5-(N-propyl-N-sulfopropyl-amino)-phenol; ISE = ion selective electrode.

*Table 4. Imprecision of the Olympus AU 5200 Analyser. Within-run coefficients of variation are median values of three series.*

| | | | Between days ($N$ = 15 days) | | | Within-run ($N$ = 20) |
|---|---|---|---|---|---|---|
| Analyte | Control material | Assigned value | Found value | Recovery range | CV % | CV % |
| *Enzymes* | | | | | | |
| Alkaline phosphatase | Monitrol 1 | 70 | 76 | 71–84 | 4·3 | 0·6 |
| (U/l) | Monitrol 2 | 293 | 317 | 304–332 | 2·7 | 0·5 |
| | Precinorm U | 271 | 282 | 267–293 | 2·1 | 0·9 |
| | Precipath U | 316 | 344 | 331–357 | 2·3 | 1·0 |
| | Qualitrol L | 168 | 142 | 135–155 | 3·3 | 0·6 |
| | Qualitrol N | 273 | 253 | 245–267 | 2·2 | 0·8 |
| | Qualitrol H | 397 | 348 | 335–370 | 2·4 | 0·5 |
| Alanine aminotransferase | Monitrol 1 | 25 | 24 | 23–25 | 2·4 | 1·6 |
| (U/l) | Monitrol 2 | 83 | 73 | 70–74 | 1·5 | 0·5 |
| | Precinorm U | 30 | 25 | 24–25 | 2·1 | 1·8 |
| | Precipath U | 63 | 53 | 52–55 | 1·5 | 0·4 |
| | Qualitrol L | 23 | 17 | 16–17 | 2·5 | 0·1 |
| | Qualitrol N | 31 | 22 | 21–23 | 3·1 | 2·2 |
| | Qualitrol H | 104 | 82 | 80–83 | 1·3 | 0.8 |
| Amylase | Monitrol 1 | | 41 | 36–48 | 10·2 | 2·0 |
| (U/l) | Monitrol 2 | | 149 | 133–156 | 3·1 | 0·7 |
| | Precinorm U | | 63 | 62–65 | 1·3 | 1·2 |
| | Precipath U | | 120 | 116–124 | 1·5 | 0·8 |
| | Qualitrol L | | 41 | 38–44 | 2·0 | 1·4 |
| | Qualitrol N | | 59 | 58–61 | 3·8 | 0·8 |
| | Qualitrol H | | 221 | 215–227 | 1·4 | 0·5 |
| Aspartate aminotransferase | Monitrol 1 | 32 | 32 | 28–34 | 4·6 | 1·6 |
| (U/l) | Monitrol 2 | 76 | 73 | 67–74 | 2·4 | 0·6 |
| | Precinorm U | 26 | 22 | 21–23 | 2·3 | 1·0 |
| | Precipath U | 60 | 52 | 49–53 | 2·2 | 0·8 |
| | Qualitrol L | 22 | 18 | 17–21 | 4·0 | 2·6 |
| | Qualitrol N | 45 | 38 | 37–39 | 1·4 | 1·8 |
| | Qualitrol H | 99 | 85 | 81–87 | 1·9 | 0·6 |
| Creatine phosphokinase | Monitrol 1 | 56 | 56 | 44–62 | 10·2 | 1·5 |
| (U/l) | Monitrol 2 | 147 | 150 | 121–179 | 9·5 | 0·8 |
| | Precinorm U | 114 | 95 | 80–113 | 7·9 | 0·7 |
| | Precipath U | 207 | 169 | 144–185 | 7·3 | 1·1 |
| | Qualitrol L | 44 | 37 | 23–48 | 15·7 | 2·2 |
| | Qualitrol N | 90 | 81 | 71–93 | 6·1 | 0·6 |
| | Qualitrol H | 200 | 145 | 88–189 | 14·2 | 0·7 |
| g-Glutamyltransferase | Monitrol 1 | 20 | 19 | 19–20 | 2·6 | 1·6 |
| (U/l) | Monitrol 2 | 46 | 43 | 43–45 | 1·5 | 0·7 |
| | Precinorm U | 38 | 36 | 35–38 | 2·2 | 1·6 |
| | Precipath U | 107 | 107 | 103–109 | 1·3 | 0·7 |
| | Qualitrol L | 15 | 14 | 14–15 | 3·0 | 3·3 |
| | Qualitrol N | 19 | 19 | 18–20 | 2·3 | 2·3 |
| | Qualitrol H | 82 | 77 | 75–79 | 1·4 | 0·7 |
| Lactate dehydrogenase | Monitrol 1 | 196 | 193 | 178–204 | 3·1 | 1·0 |
| (U/l) | Monitrol 2 | 319 | 308 | 294–324 | 2·5 | 0·5 |
| | Precinorm U | 175 | 148 | 135–156 | 3·2 | 1·3 |
| | Precipath U | 266 | 229 | 217–242 | 2·7 | 0·9 |
| | Qualitrol L | 129 | 110 | 104–121 | 3·0 | 1·5 |
| | Qualitrol N | 306 | 251 | 239–259 | 2·5 | 0·6 |
| | Qualitrol H | 548 | 447 | 426–461 | 2·2 | 0·4 |
| *Substrates* | | | | | | |
| Albumin | Monitrol 1 | 4·3 | 3·5 | 3·4–3·6 | 1·7 | 1·4 |
| (g/dl) | Monitrol 2 | 3·3 | 2·5 | 2·4–2·6 | 1·7 | 1·7 |
| | Precinorm U | 3·5 | 2·6 | 2·5–2·8 | 2·3 | 1·7 |
| | Precipath U | 3·3 | 2·5 | 2·3–2·6 | 2·6 | 1·2 |
| | Qualitrol L | 2·1 | 1·8 | 1·7–1·9 | 2·5 | 2·2 |
| | Qualitrol N | 4·7 | 4·2 | 4·1–4·3 | 1·4 | 1·4 |
| | Qualitrol H | 4·1 | 3·9 | 3·8–4·1 | 1·9 | 1·1 |

*Table 4 (continued).*

| | | | Between days ($N$ = 15 days) | | | Within-run ($N$ = 20) |
|---|---|---|---|---|---|---|
| Analyte | Control material | Assigned value | Found value | Recovery range | CV % | CV % |
| Total bilirubin (mg/dl) | Monitrol 1 | 1·3 | 1·3 | 1·1–1·3 | 4·6 | 1·7 |
| | Monitrol 2 | 4·6 | 4·6 | 4·5–5·0 | 2·7 | 1·5 |
| | Precinorm U | 2·1 | 2·0 | 1·7–2·1 | 5·5 | 2·3 |
| | Precipath U | 4·4 | 4·5 | 4·0–4·8 | 4·1 | 1·5 |
| | Qualitrol L | 1·5 | 1·6 | 1·5–1·6 | 2·5 | 1·4 |
| | Qualitrol N | 1·2 | 1·3 | 1·1–1·4 | 5·2 | 2·9 |
| | Qualitrol H | 4·5 | 5·0 | 4·9–5·2 | 1·7 | 1·8 |
| Calcium (mmol/l) | Monitrol 1 | 2·3 | 2·3 | 2·3–2·3 | 1·0 | 0·7 |
| | Monitrol 2 | 3·2 | 3·2 | 3·1–3·2 | 1·2 | 0·7 |
| | Precinorm U | 2·6 | 2·7 | 2·6–2·7 | 1·3 | 1·0 |
| | Precipath U | 3·5 | 3·5 | 3·4–3·6 | 1·4 | 0·5 |
| | Qualitrol L | 1·3 | 1·4 | 1·3–1·4 | 1·5 | 0·9 |
| | Qualitrol N | 2·8 | 2·7 | 2·7–2·9 | 2·3 | 0·7 |
| | Qualitrol H | 3·1 | 3·1 | 3·1–3·2 | 1·2 | 0·8 |
| Cholesterol (mg/dl) | Monitrol 1 | 184 | 176 | 172–184 | 1·7 | 0·6 |
| | Monitrol 2 | 124 | 114 | 112–118 | 1·3 | 0·6 |
| | Percinorm U | 106 | 95 | 92–98 | 1·4 | 0·7 |
| | Precipath U | 125 | 115 | 113–120 | 1·6 | 0·7 |
| | Qualitrol L | 117 | 107 | 102–112 | 1·8 | 0·5 |
| | Qualitrol N | 114 | 107 | 104–110 | 1·4 | 0·7 |
| | Qualitrol H | 233 | 218 | 213–224 | 1·3 | 0·5 |
| Creatinine Jaffe (mg/dl) | Monitrol 1 | 1·2 | 1·1 | 1·0–1·2 | 4·1 | 3·1 |
| | Monitrol 2 | 5·0 | 5·1 | 4·9–5·3 | 1·6 | 1·5 |
| | Precinorm U | 1·7 | 1·9 | 1·8–2·3 | 6·2 | 3·2 |
| | Precipath U | 3·3 | 3·5 | 3·2–3·7 | 2·7 | 1·9 |
| | Qualitrol L | 1·2 | 1·1 | 1·0–1·2 | 5·1 | 4·7 |
| | Qualitrol N | 1·0 | 0·9 | 0·8–1·0 | 6·1 | 4·0 |
| | Qualitrol H | 7·1 | 6·6 | 6·4–6·7 | 1·3 | 1·4 |
| Creatinine enz. (mg/dl) | Monitrol 1 | 1·1 | 1·1 | 1·1–1·2 | 1·1 | 1·5 |
| | Monitrol 2 | 5·7 | 6·2 | 5·6–6·4 | 2·6 | 1·0 |
| | Precinorm U | 1·9 | 1·9 | 1·4–2·0 | 6·9 | 1·5 |
| | Precipath U | 3·9 | 4·1 | 3·7–4·2 | 3·2 | 0·8 |
| | Qualitrol L | 1·2 | 1·3 | 0·9–1·4 | 8·9 | 1·5 |
| | Qualitrol N | 0·8 | 0·8 | 0·8–0·9 | 1·8 | 1·7 |
| | Qualitrol H | 7·2 | 8·1 | 7·7–8·4 | 2·0 | 0·5 |
| Glucose (mg/dl) | Monitrol 1 | 75 | 88 | 84–91 | 2·8 | 0·6 |
| | Monitrol 2 | 245 | 254 | 239–269 | 4·0 | 0·7 |
| | Precinorm U | 114 | 120 | 114–123 | 1·6 | 1·0 |
| | Precipath U | 236 | 249 | 242–257 | 1·6 | 0·6 |
| | Qualitrol L | 41 | 45 | 40–44 | 5·3 | 1·4 |
| | Qualitrol N | 95 | 102 | 98–109 | 2·4 | 0·7 |
| | Qualitrol H | 206 | 207 | 200–213 | 1·7 | 0·6 |
| Inorganic phosphate (mg/dl) | Monitrol 1 | 3·4 | 3·6 | 3·5–3.7 | 1·3 | 1·8 |
| | Monitrol 2 | 6·9 | 7·2 | 6·9–7·4 | 2·0 | 0·5 |
| | Precinorm U | 5·2 | 5·5 | 5·2–5·6 | 2·1 | 1·0 |
| | Precipath U | 6·0 | 6·3 | 6·0–6·5 | 2·0 | 0·8 |
| | Qualitrol L | 4·0 | 4·1 | 3·9–4·3 | 2·1 | 1·8 |
| | Qualitrol N | 2·6 | 2·3 | 2·1–2·3 | 2·9 | 1·5 |
| | Qualitrol H | 8·1 | 8·8 | 8·5–9·1 | 2·1 | 0·6 |
| Iron (ug/dl) | Monitrol 1 | 108 | 101 | 97–109 | 2·7 | 2·5 |
| | Monitrol 2 | 190 | 214 | 205–239 | 4·1 | 0·7 |
| | Precinorm U | 120 | 115 | 112–120 | 1·8 | 1·2 |
| | Precipath U | 136 | 129 | 126–132 | 1·5 | 1·0 |
| | Qualitrol L | 84 | 84 | 75–95 | 6·4 | 2·3 |
| | Qualitrol N | 211 | 190 | 184–207 | 3·1 | 0·6 |
| | Qualitrol H | 181 | 175 | 169–185 | 2·3 | 1·1 |

*Table 4 (continued).*

| Analyte | Control material | Assigned value | Between days ($N$ = 15 days) Found value | Recovery range | CV % | Within-run ($N$ = 20) CV % |
|---|---|---|---|---|---|---|
| Total protein (g/dl) | Monitrol 1 | 6·8 | 7·0 | 6·8–7·2 | 1·4 | 0·6 |
| | Monitrol 2 | 5·1 | 5·1 | 5·0–5·3 | 1·4 | 0·9 |
| | Precinorm U | 5·0 | 5·0 | 4·9–5·1 | 1·2 | 1·0 |
| | Precipath U | 4·9 | 4·9 | 4·8–5·0 | 1·2 | 0·8 |
| | Qualitrol L | 4·7 | 4·7 | 4·5–4·8 | 1·9 | 1·0 |
| | Qualitrol N | 8·7 | 8·7 | 7·8–8·1 | 1·2 | 0·5 |
| | Qualitrol H | 8·9 | 8·9 | 8·9–9·3 | 1·0 | 0·5 |
| Triglyzerides (mg/dl) | Monitrol 1 | 88 | 80 | 77–82 | 1·6 | 0·9 |
| | Monitrol 2 | 184 | 167 | 160–173 | 1·6 | 0·9 |
| | Precinorm U | 105 | 90 | 86–93 | 1·8 | 0·9 |
| | Precipath U | 136 | 114 | 103–118 | 2·6 | 0·9 |
| | Qualitrol L | 93 | 79 | 76–81 | 1·6 | 1·2 |
| | Qualitrol N | 128 | 107 | 102–109 | 1·7 | 0·5 |
| | Qualitrol H | 251 | 209 | 202–215 | 1·7 | 0·4 |
| Urea (mg/dl) | Monitrol 1 | 32 | 31 | 30–33 | 2·5 | 1·9 |
| | Monitrol 2 | 108 | 109 | 106–114 | 1·9 | 2·4 |
| | Precinorm U | 57 | 56 | 55–59 | 2·1 | 1·8 |
| | Precipath U | 151 | 157 | 152–161 | 1·9 | 2·2 |
| | Qualitrol L | 16 | 15 | 14–16 | 3·5 | 3·3 |
| | Qualitrol N | 38 | 37 | 35–39 | 2·8 | 1·4 |
| | Qualitrol H | 168 | 174 | 169–179 | 1·6 | 1·1 |
| Uric acid (mg/dl) | Monitrol 1 | 5·3 | 5·2 | 5·0–5·4 | 2·0 | 1·2 |
| | Monitrol 2 | 8·3 | 8·2 | 7·6–8·6 | 2·4 | 0·7 |
| | Precinorm U | 5·9 | 4·9 | 4·8–5·2 | 2·1 | 1·2 |
| | Precipath U | 10·2 | 10·4 | 10·1–10·7 | 1·7 | 1·1 |
| | Qualitrol L | 3·6 | 3·3 | 3·1–3·4 | 1·9 | 1·7 |
| | Qualitrol N | 6·9 | 7·0 | 6·7–7·2 | 1·8 | 1·0 |
| | Qualitrol H | 10·2 | 10·0 | 9·5–10·3 | 2·0 | 1·6 |
| *Electrolytes* | | | | | | |
| Sodium (mmol/l) | Monitrol 1 | 139 | 140 | 136–145 | 2·5 | 0·3 |
| | Monitrol 2 | 111 | 112 | 108–117 | 2·3 | 0·9 |
| | Precinorm U | 123 | 124 | 118–129 | 2·2 | 0·7 |
| | Precipath U | 137 | 141 | 137–147 | 2·6 | 0·4 |
| | Qualitrol L | 116 | 120 | 114–122 | 2·3 | 0·4 |
| | Qualitrol N | 154 | 155 | 150–159 | 2·4 | 0·3 |
| | Qualitrol H | 161 | 164 | 159–167 | 2·3 | 0·3 |
| Potassium (mmol/l) | Monitrol 1 | 4·40 | 4·41 | 4·30–4·60 | 1·8 | 0·6 |
| | Monitrol 2 | 6·65 | 6·81 | 6·60–7·10 | 2·3 | 1·5 |
| | Precinorm U | 4·64 | 4·73 | 4·70–4·90 | 1·5 | 0·8 |
| | Precipath U | 6·91 | 7·06 | 6·90–7·30 | 1·7 | 0·5 |
| | Qualitrol L | 2·28 | 2·32 | 2·20–2·40 | 1·7 | 1·2 |
| | Qualitrol N | 5·25 | 5·45 | 5·20–5·60 | 1·9 | 0·5 |
| | Qualitrol H | 6·50 | 6·69 | 6·40–6·80 | 2·0 | 0·4 |
| Chloride (mmol/l) | Monitrol 1 | 109 | 110 | 107–115 | 1·9 | 0·4 |
| | Monitrol 2 | 90 | 90 | 89–93 | 1·7 | 0·5 |
| | Precinorm U | 77 | 81 | 78–84 | 1·6 | 0·5 |
| | Precipath U | 119 | 124 | 121–130 | 1·9 | 0·4 |
| | Qualitrol L | 84 | 86 | 82–90 | 1·5 | 0·5 |
| | Qualitrol N | 94 | 95 | 93–100 | 1·7 | 0·4 |
| | Qualitrol H | 120 | 118 | 114–119 | 1·6 | 0·4 |

several times. If carry-over had occurred, samples immediately following the groups with high activity should have shown elevated activities.

### *Correlation study*

Approximately 250 fresh human sera from daily routine samples were analysed simultaneously in the AU 5200 and in the AU 5000. In addition, sodium, potassium and chloride concentrations in the same samples were measured in a Hitachi 747 analyser equipped with a ISE unit. Care was taken to include pathological analyte levels.

### *Assessment of drift effects*

Drift effects were studied using seven commercial control sera. The sera were dissolved before starting the drift experiment and kept at 4°C until use. For determination of alkaline phosphatase, creatine phosphokinase and bilirubin the sera were freshly reconstituted every hour. Five measurements of each control serum were carried out every 60 min for 9 h. Prior to starting a series of measurements the reagent lines were flushed with fresh reagent.

In order to detect short-term variations or sudden changes, a second experiment was conducted. Chemistry

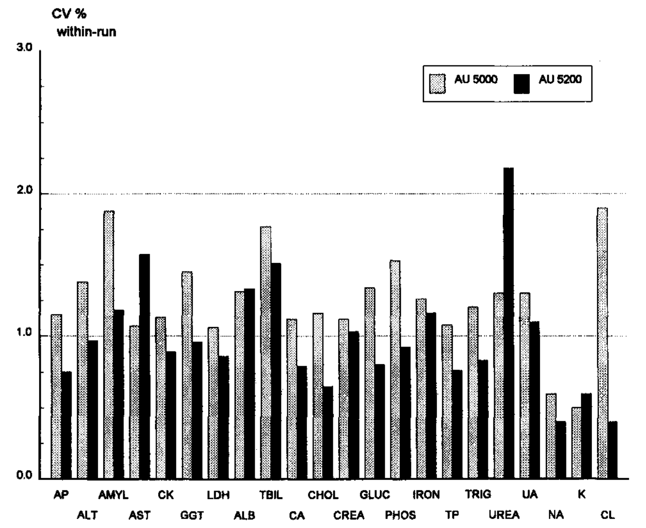

*Figure 1 (a). Imprecision within-run on both the AU 5200 and the comparison instrument. The bars plotted in this figure represent median CV values of the data in table 4.*

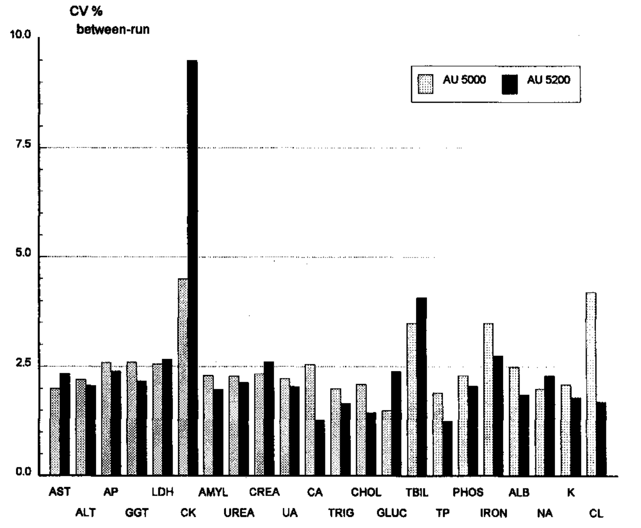

*Figure 1 (b). Imprecision between-run on both the AU 5200 and the comparison instrument. The bars plotted in this figure represent median CV values of the data in table 4.*

*Table 5. Recovery of assigned values in quality-control materials. The minimum and maximum of the assigned values are shown for all tested paramaters. The median, 10th, 25th, 75th and 90th percentiles of the found values are expressed as percentages of the assigned values.*

| | | Assigned | | Found (%) | | | | |
|---|---|---|---|---|---|---|---|---|
| Analyte | *N* | Minimum | Maximum | Median | 10th P. | 25th P. | 75th P. | 90th P. |
| *Enzymes* | | | | | | | | |
| Alkaline phosphatase | 22 | 47 | 423 | 103 | 85 | 88 | 107 | 112 |
| Alanine aminotransferase | 22 | 16 | 104 | 85 | 78 | 83 | 88 | 94 |
| Amylase | 11 | 20 | 173 | 95 | 91 | 92 | 100 | 111 |
| Aspartate aminotransferase | 22 | 10 | 99 | 87 | 82 | 84 | 94 | 104 |
| Creatine phosphokinase | 22 | 22 | 229 | 85 | 74 | 82 | 96 | 100 |
| g-Glutamyl-transferase | 22 | 10 | 110 | 101 | 94 | 98 | 106 | 110 |
| Lactate dehydrogenase | 22 | 123 | 548 | 92 | 86 | 87 | 99 | 102 |
| *Substrates* | | | | | | | | |
| Albumin | 25 | 2·1 | 4·7 | 83 | 76 | 78 | 88 | 94 |
| Total bilirubin | 27 | 0·7 | 7·3 | 108 | 98 | 102 | 114 | 118 |
| Calcium | 29 | 1·3 | 3·5 | 101 | 97 | 100 | 102 | 104 |
| Cholesterol | 27 | 86 | 241 | 94 | 91 | 93 | 96 | 98 |
| Creatinine Jaffe | 23 | 0·4 | 6·9 | 105 | 97 | 100 | 102 | 108 |
| Creatinine enz. | 19 | 0·7 | 7·1 | 108 | 98 | 101 | 112 | 113 |
| Glucose | 23 | 41 | 305 | 105 | 99 | 103 | 107 | 108 |
| Inorganic phosphate | 27 | 2·0 | 8·4 | 105 | 93 | 99 | 109 | 110 |
| Iron | 27 | 68 | 268 | 97 | 86 | 93 | 100 | 102 |
| Total protein | 29 | 4·1 | 8·9 | 103 | 99 | 101 | 106 | 107 |
| Triglyzerides | 27 | 50 | 251 | 90 | 83 | 87 | 96 | 99 |
| Urea | 29 | 14 | 169 | 99 | 94 | 96 | 102 | 105 |
| Uric acid | 27 | 2·6 | 10·8 | 101 | 95 | 100 | 105 | 107 |
| *Electrolytes* | | | | | | | | |
| Sodium | 25 | 111 | 163 | 99 | 98 | 99 | 100 | 101 |
| Potassium | 25 | 2·3 | 7·3 | 100 | 98 | 100 | 101 | 103 |
| Chloride | 19 | 84 | 124 | 101 | 96 | 97 | 103 | 104 |

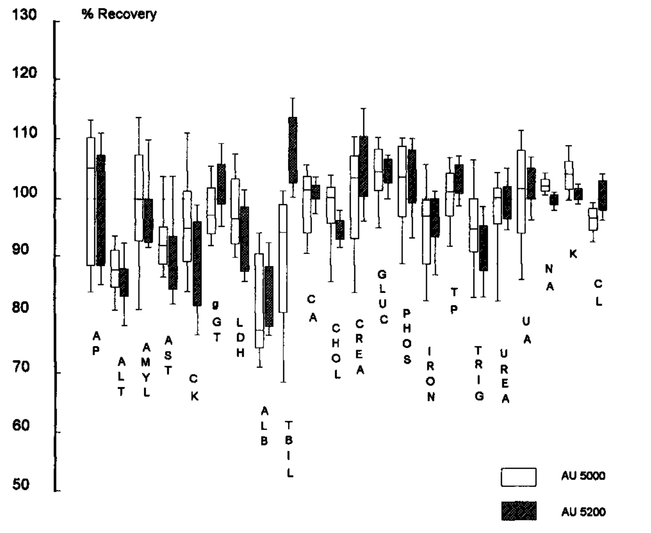

*Figure 2. Recovery of assigned values in 32 commercial control materials on both the AU 5200 and the comparison instrument. The boxes represent the 25th, 75th percentiles and medians, the bars the 10th and 90th percentiles of the recovery ratios in percentages.*

and ISE tests were performed continuously over a period of 6 h. Therefore a serum pool was prepared, filtered and stirred on a magnetic stirrer at 4°C. Approximately 1800 aliquots of this serum pool were taken and analysed in the AU 5200 immediately. Blank readings and calibration of the instrument were performed only once at the start of the experiments.

### Results

### *Imprecision*

Data on within-run and between-day imprecision are summarized in table 4. The values are medians from three independent measurement series for seven control sera. Figures 1(*a*) and 1(*b*) show coefficients of variation for all tests on both the AU 5200 and AU 5000 analysers. The CV values in these figures are again medians taken across the CV values in table 4 and corresponding values obtained from measurements on the AU 5000 analyser.

As indicated in figure 1(*a*), 21 of 23 evaluated tests on the AU 5200 analyser yielded a median within-run imprecision well below 2·0%; urea showed a median imprecison of 2·1%. Creatinine, measured with the kinetic Jaffe reaction method on the AU 5200, yielded rather high CV values from 1·5% to 4·7% (not shown in figures 1(*a*) and 1(*b*).

The electrolyte measurements on the AU 5200 analyser showed a remarkably good precision. Slightly lower median CV values for the imprecision in series were found for 18 of 22 tests on the AU 5200, compared with the AU 5000 analyser.

The median between-day CV values were below 3·0% for 19 out of 23 tests on the AU 5200 analyser and were well below 5·0% for all tests, with the exception of the CPK activity measurement which showed CV values from 6·1% to 15·7%.

The majority of all tests gave lower imprecision from day to day on the AU 5200 analyser compared to those on the AU 5000.

The overall precision in series and from day to day on both analysers could be rated as very good, with the AU 5200 performing slightly better than the AU 5000 analyser.

*Table 6. Limits of the analytical range.*

| Analyte | Unit | Measured up to | Upper limit of linearity Found | Upper limit of linearity Declared |
|---|---|---|---|---|
| *Enzymes* | | | | |
| Alkaline phosphatase | U/l | 3300[3] | 3300 | 1600 |
| Alanine aminotransferase | U/l | 700 | 600 | 400 |
| Amylase | U/l | 1000[3] | 1000 | 1350 |
| Aspartate aminotransferase | U/l | 500 | 500 | 450 |
| Creatine phosphokinase | U/l | 1200 | 1200 | 1100 |
| g-Glutamyl-transferase | U/l | 900 | 900 | 500 |
| Lactate dehydrogenase | U/l | 2500 | 1750 | 500 |
| *Substrates* | | | | |
| Albumin | g/dl | 7·0 | 7·0 | 7·0 |
| Total bilirubin | mg/dl | 25·0 | 25·0 | 30·0 |
| Calcium | mmol/l | 7·0 | 6·0 | 5·0 |
| Cholesterol | mg/dl | 1200 | 1000 | 750 |
| Creatinine enz.[1] | mg/dl | 30 | 30 | 20 |
| Glucose[2] | mg/dl | 1000 | 1000 | 700 |
| Inorganic phosphate | mg/dl | 22·5 | 22·5 | 20 |
| Iron | ug/dl | 1000 | 1000 | 1000 |
| Total protein | g/dl | 12·0 | 12·0 | 12·0 |
| Triglyzerides | mg/dl | 1200 | 1000 | 1000 |
| Urea | mg/dl | 400 | 375 | 300 |
| Uric Acid | mg/dl | 33·0 | 33·0 | 20·0 |

[1] Creatinine PAP (Merck); [2] Hexokinase method; [3] Extinction beyond DOD range.

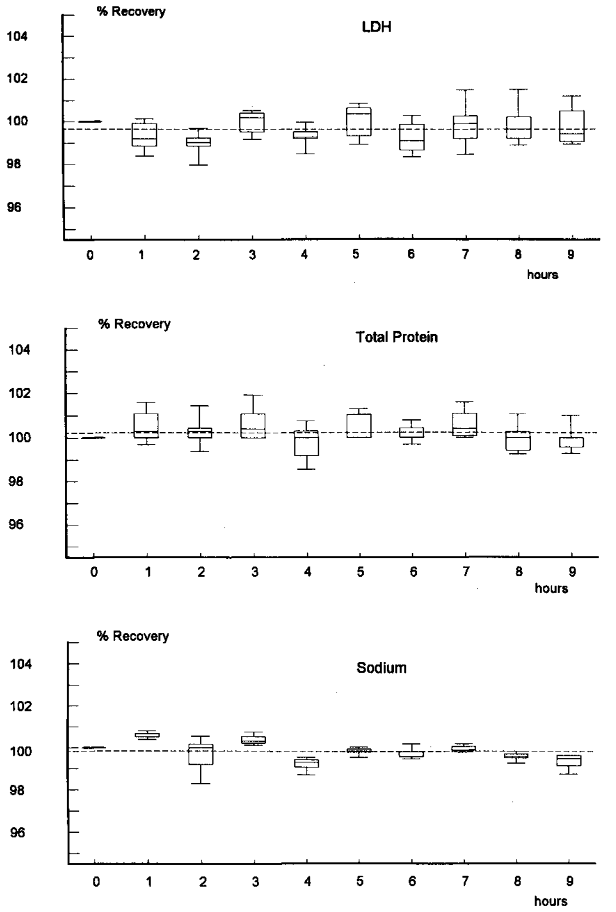

*Figure 3 (a). Drift during 9 h for three representative analytes measured in seven control sera. The medians, 10th, 25th, 75th and 90th percentiles of the recovery ratios in relation to the initial measurements are shown as box-plots.*

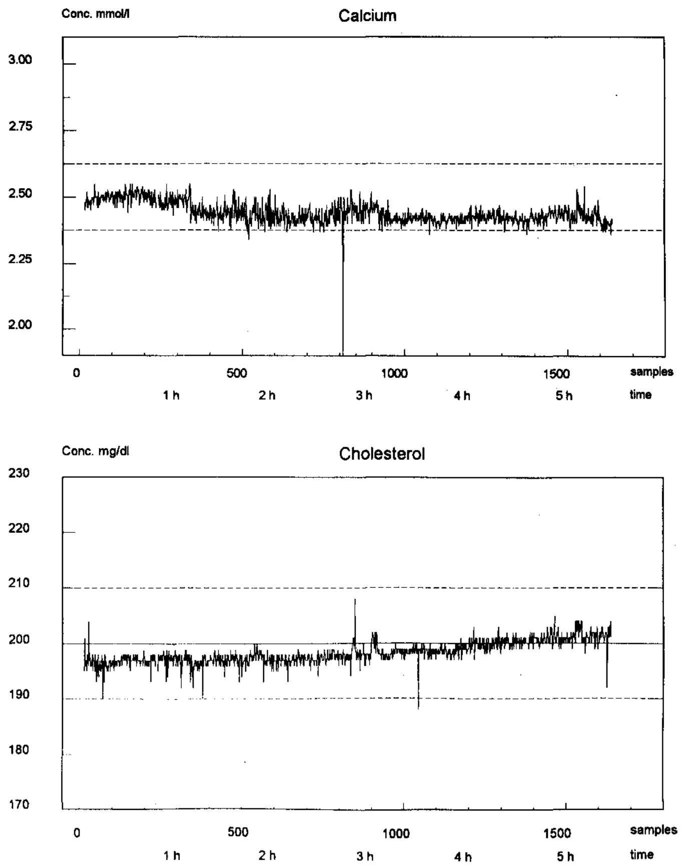

*Figure 3 (b). Drift during 6 h of continuous operation. Approximately 1800 measurements of all analytes were performed.*

### *Assessment of accuracy*

The recovered values of all analytes in 32 commercial control sera were expressed as percentages of the assigned values. The medians, the 10th, 25th, 75th and 90th percentiles of the respective values are shown in table 5 and in figure 2. Fourteen of 23 tests yielded median recoveries within the 5% range; five tests were in the range between 5% and 10% (lactate dehydrogenase, bilirubin, cholesterol, triglyzerides and creatinine measured enzymatically); and three tests were in the range between 10% and 15% (alanine aminotransferase, creatine phosphokinase).

The low recoveries of some enzyme activity tests may be due to the conversion of the values measured to corresponding values at 25 °C. These conversion factors are based upon human serum, whereas most commercial control materials are essentially non-human or spiked with non-human analytes. The low recoveries of albumin (83%), cholesterol and triglyzerides were caused by calibration errors. As a consequence of this evaluation the calibrator values for these tests were reassigned by the manufacturer.

### *Linearity*

Both the linearities found, and the linearity ranges given by the manufacturers, are shown in table 6. The linearities found were better than the manufacturers' for all tests, with the exception of amylase. The transaminases showed limited linearities with an upper range of approximately 500 U/I, whereas cholesterol and triglyceride measurement is possible up to 1000 mg/dl.

### *Carry-over*

A potential carry-over of sample residue resulting from an insufficient washing of the cuvettes was investigated by measuring creatine phospokinase activity (27 U/l in a serum pool) in cuvettes which had been used for the determination of the same analyte with a very high activity (approximately 1500 U/l). The results were compared with measurements of low activity in cuvettes which had also been used for measurements of low activity. Figure 4(*a*) shows that there was no detectable difference. Sample carry-over due to incomplete washing of the probe needle and the mixer blades was investigated by alternating two serum pools with high and low creatine phosphokinase activity; the samples which immediately followed the groups with high activity did not show elevated activities (figure 4(*b*)). In conclusion, no specimen-related carry-over could be detected.

### *Method comparison with patient samples*

The Olympus AU 5200 series analyser was compared with an Olympus AU 5000 analyser and a Hitachi 747 analyser (ISE measurements only). The statistical evaluation was performed according to the method of Passing and Bablock [3]. The numerical results are given in

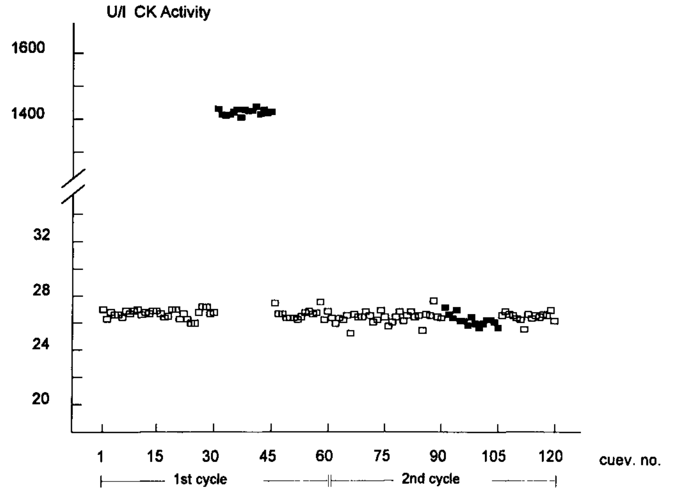

*Figure 4 (a). Specimen related carry-over due to incomplete cuvette washing: creatine phosphokinase measurements of 120 samples of two serum pools with a low and a high activity in two complete cuvette wheel cycles. During the first cycle samples with a high activity (approximately 1500 U/l) were measured in cuvettes 31 to 45. In the same cuvettes samples with a low activity were measured during the second cycle (indicated by filled squares).*

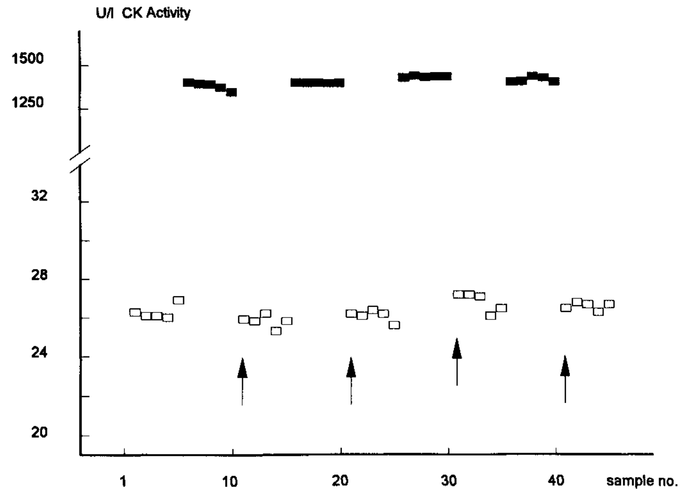

*Figure 4 (b). Specimen related carry-over due to incomplete washing of the probe needles and mixer blades. Groups of five samples with low and high activity of creatine phosphokinase were alternated several times. The arrow bars mark the first samples with low activity immediately following a sequence of samples with high activity.*

table 7, and scatterplots are shown in figure 5. The correlations were close for all enzyme and substrate tests. Good agreement was found between flame photometry (AU 5000) and ISE measurement (Hitachi 747) for potassium. Sodium (flame photometry and ISE) and chloride measurement (mercury thiocyanate) showed lower coefficients of correlation.

Alkaline phosphatase, creatine phosphokinase, g-glutamyltransferase, aspartate aminotransferase, alanine aminotransferase, lactate dehydrogenase, calcium, creatinine (measured enzymatically), inorganic phosphate, total bilirubin, triglyzerides, urea, uric acid, potassium (flame photometry and ISE), sodium (flame photometry) and chloride (mercury thiocyanate method and ISE) did not show a significant deviation from the factor 1 for the slope or from the constant 0 for the intercept. Albumin, cholesterol, glucose (hexokinase method), iron, total protein and sodium (ISE) measurements showed small, but significant, deviation of slope or intercept. Amylase measurements on both analysers correlated well, but are not comparable without data transformation due to different test methods with different amylase substrates.

Possible reason for the discrepancies in the albumin and cholesterol assays could be incorrect calibrator values (see above), or, in the case of iron, to different test methodologies (see table 2).

### *Drift*

The results of the drift experiments are displayed in figures 3(*a*) (measurements every hour for 9 h) and 3(*b*) (continuous measurements for 6 h). A continuous trend either increasing or decreasing could not be detected for any of the parameters tested. Median deviations from the initial values did not exceed 5% over 9 h for any test. The electrolyte measurements showed exceptionally low deviations from the starting value, indicating a very stable ISE system. The second drift experiment revealed short-term fluctuations in the measurement of creatinine (Jaffe method). Statistical spectral analysis yielded a fourier frequency of about 0·05, which is equivalent to an oscillation period of 20 samples. The remaining tests did not show any periodic variations or sudden fluctuations.

### *Assessment of practicability*

An instruction period of four days was necessary to acquire the knowledge to operate the Olympus AU 5200 analyser, users of the older AU 5000 analyser can operate the new system instantly. Despite the size and capacity of the analyser, the Olympus AU 5200 is easy to run and can easily be integrated into the organization of a large laboratory. The sample feeding mechanism operates in a linear fashion, allowing continuous supply of subsequent samples. One sample which is nowadays not an aliquot, but delivered in a primary tube, passes through the instrument in 5 min and even tubes on a fully loaded sample tray are accessible after 70 min. The sample belt is in front of the instrument, which is a major advantage over the older AU 5000 series analyser where it is in the rear. The analyser can be reached from all sites and all mechanical movements can be watched.

The system-processing software for routine operation is clearly arranged; the main jobs, for example activating the analyser out of a standby status, starting measurements, washing the cuvettes and flushing the reagent lines, are presented in one menue and are initiated by one keystroke. The software contains an extensive diagnostic program, which allows a specific detection of mechanical or electronic problems.

The preanalytical preparation of the analyser consists of four steps requiring the time periods given in parenthesis: warming up to 37 °C (60 min), washing of cuvettes and

*Table 7. Method comparison studies in human sera with Olympus AU 5200 (y) and comparison instrument (x). Regression analyses (y = a + bx) were performed according to Passing and Bablock.*

| Analyte | N | r | b[1] | a[2] | median x | median y |
|---|---|---|---|---|---|---|
| *Enzymes* | | | | | | |
| Alkaline phosphatase | 223 | 1·000 | 1·03 | 0·69 | 124·0 | 129·0 |
| Alanine aminotransferase | 242 | 0·993 | 0·96 | −0·85 | 15·5 | 14·0 |
| Amylase | 227 | 0·995 | *0·33 | *0·27 | 94·0 | 32·0 |
| Aspartate aminotransferase | 242 | 0·999 | 1·00 | −1·00 | 12·0 | 11·0 |
| Creatine phosphokinase | 219 | 0·993 | 0·89 | 1·00 | 25·0 | 23·0 |
| g-Glutamyltransferase | 237 | 0·999 | 1·09 | 0·00 | 26·0 | 28·0 |
| Lactate dehydrogenase | 234 | 0·997 | 0·93 | 1·60 | 185·5 | 177·0 |
| *Substrates* | | | | | | |
| Albumin | 239 | 0·977 | 0·93 | *−0·19 | 3·89 | 3·43 |
| Total bilirubin | 231 | 0·999 | 1·11 | −0·06 | 0·70 | 0·70 |
| Calcium | 239 | 0·963 | 1·00 | 0·03 | 2·34 | 2·37 |
| Cholesterol | 234 | 0·997 | 0·95 | *−1·54 | 186·5 | 174·5 |
| Creatinine enz. | 243 | 0·997 | 1·00 | 0·00 | 1·10 | 1·10 |
| Glucose | 243 | 0·997 | 0·99 | *1·06 | 114·0 | 115·0 |
| Inorganic phosphate | 233 | 0·991 | 0·98 | 0·08 | 3·70 | 3·80 |
| Iron | 216 | 0·991 | *1·02 | −5·86 | 87·5 | 80·5 |
| Total protein | 231 | 0·971 | *1·04 | *−0·14 | 6·90 | 6·90 |
| Triglyzerides | 235 | 0·999 | 0·93 | 1·69 | 143·0 | 133·0 |
| Urea | 238 | 0·998 | 1·04 | −1·74 | 42·0 | 41·0 |
| Uric acid | 235 | 0·992 | 1·00 | −0·20 | 5·70 | 5·60 |
| *Electrolytes* | | | | | | |
| Sodium<br>AU 5200 ISE (y)<br>AU 5000 Flame (x) | 291 | 0·834 | 1·00 | −3.00 | 149·0 | 147·0 |
| Sodium<br>AU 5200 ISE (y)<br>Hit 747 ISE (x) | 299 | 0·879 | *0·90 | *11·10 | 151·0 | 147·0 |
| Potassium<br>AU 5200 ISE (y)<br>AU 5000 Flame (x) | 287 | 0·956 | 1·00 | −0·10 | 4·70 | 4·50 |
| Potassium<br>AU 5200 ISE (y)<br>Hit 747 ISE (x) | 298 | 0·989 | 1·00 | −0·10 | 4·65 | 4·50 |
| Chloride<br>AU 5200 ISE (y)<br>AU 5000 Hg (x) | 299 | 0·864 | 0·85 | 14·00 | 109·0 | 108·0 |
| Chloride<br>AU 5200 ISE (y)<br>Hit 747 ISE (x) | 297 | 0·949 | 0·84 | 16·48 | 110·0 | 108·2 |

*: [1] Significant deviation of slope b from 1·0; [2] significant deviation of intercept a from 0.

filling reagent lines (23 min), reagent blanking, calibration and running controls (20 min). Using the automatic power-on facility reduces the preanalytic phase by the time period for warming up the instrument. Thus, after 45 min, including the time needed for checking the results of calibration and control materials, the analyser is ready for routine operation.

During the evaluation period and the subsequent routine work no total shut down of the analyser occurred. The most frequent problems were rack jams on the transport belt and needle crashes. In such situations the analyser does not stop completely, but stops only the feeding mechanism. Results of tests pipetted so far can still be obtained. The most severe faults were worn out gears of the cuvette wheel motor causing a small displacement of the cuvette wheel hindering the action of the washer unit; and, a leakage in the vacuum system of the washer unit causing an incomplete draining of the cuvettes. Both problems could be attributed to some kind of material fatigue. The manufacturer has now replaced the materials for these machine parts. Another point of criticism is the badly arranged report format in which the results are printed out – there is no way of achieving patient-oriented reports.

### Conclusion

With respect to imprecision, accuracy, linearity and carry-over data, the Olympus AU 5200 analyser compared

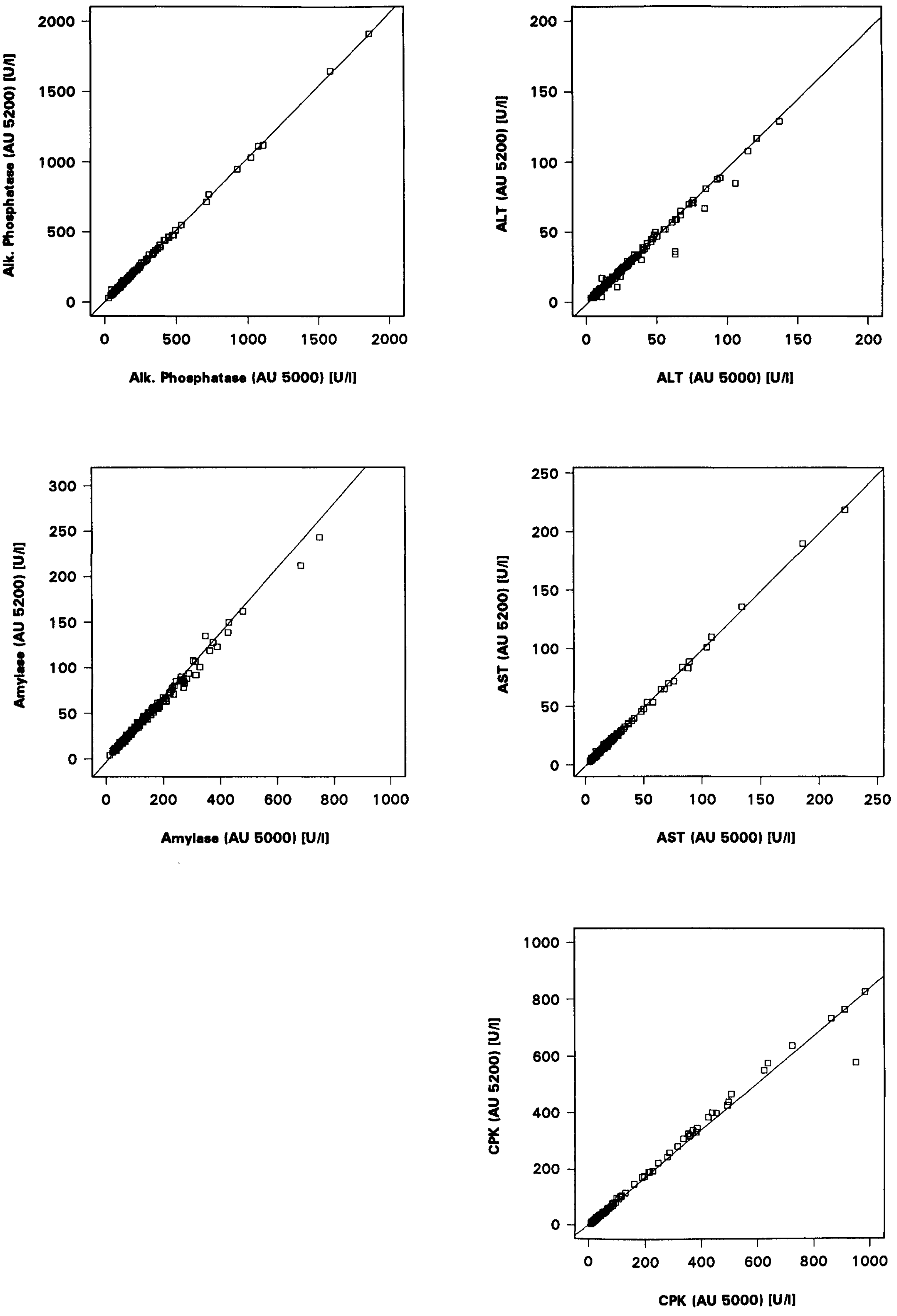

*Figure 5 (a). Graphical plots of instrument comparison.*

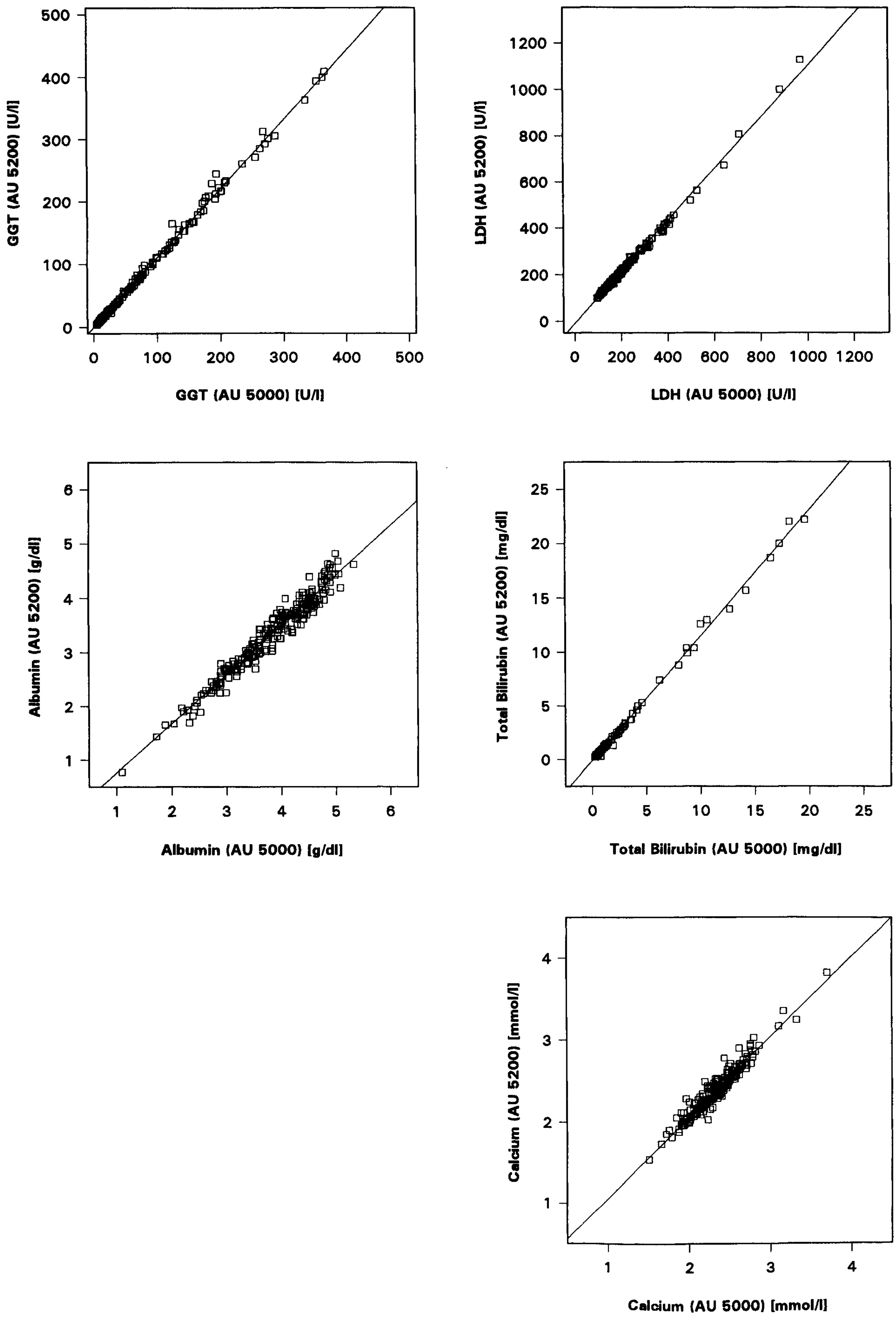

*Figure 5 (b). Graphical plots of instrument comparison.*

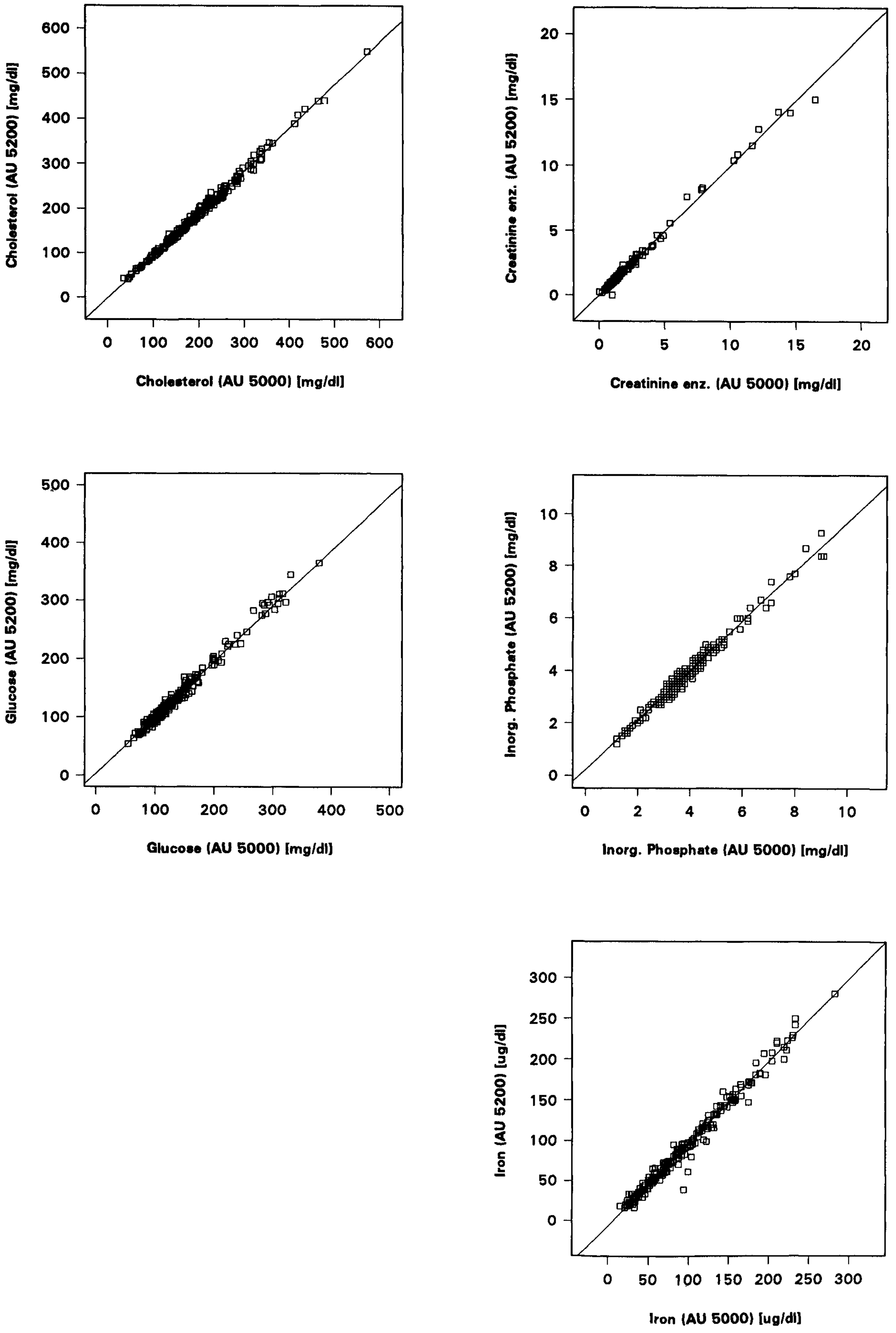

*Figure 5 (c). Graphical plots of instrument comparison.*

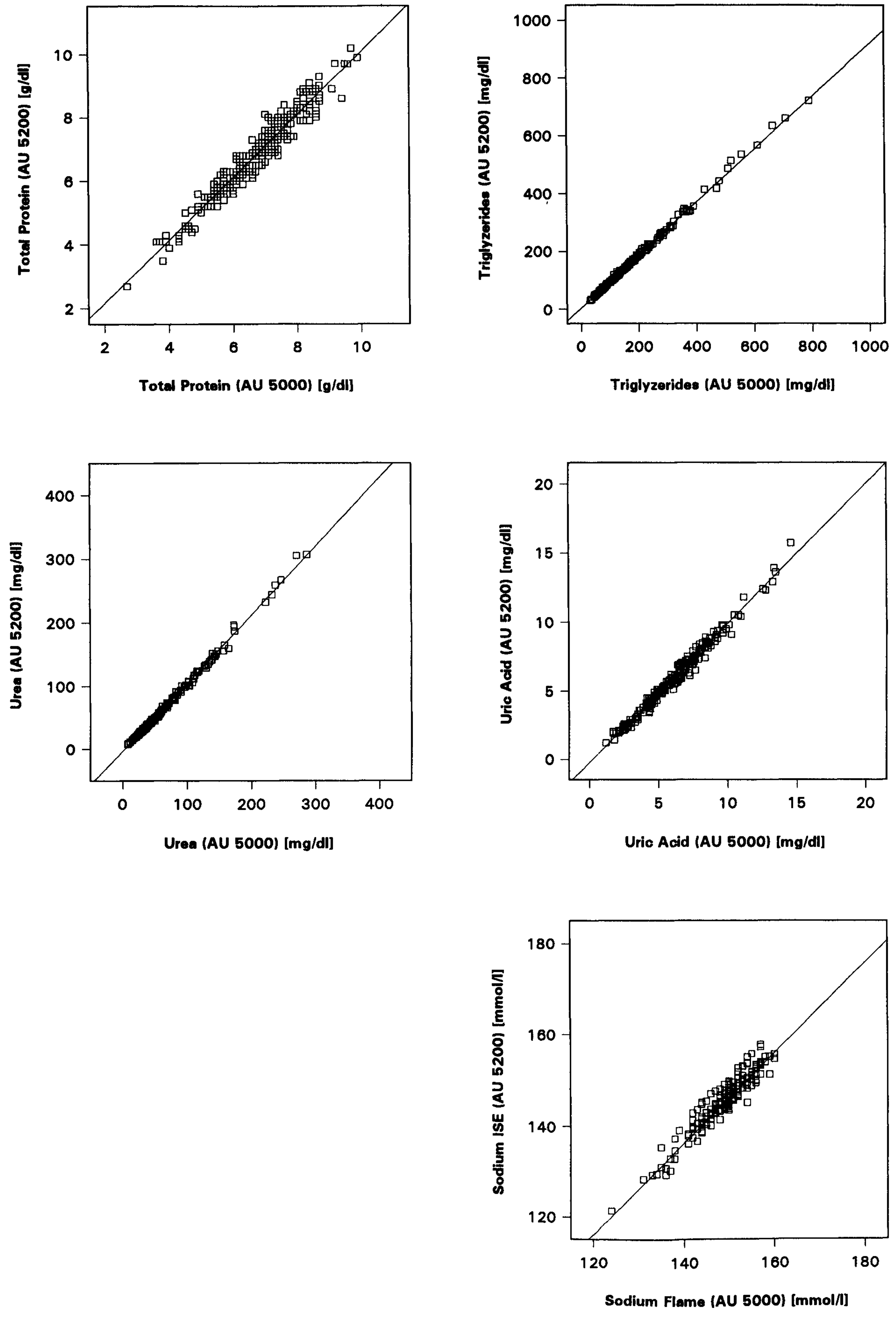

*Figure 5 (d). Graphical plots of instrument comparison.*

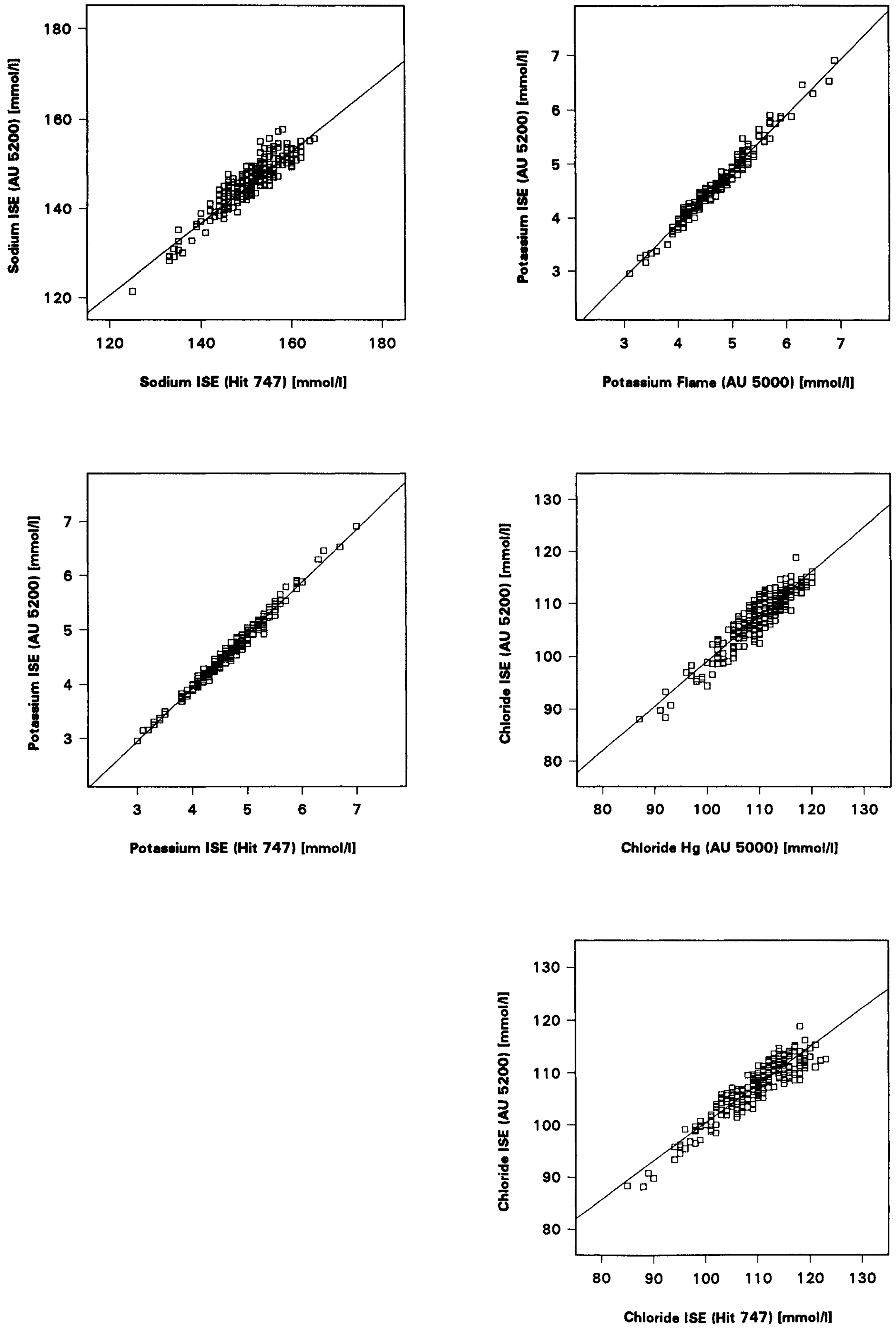

*Figure 5 (e). Graphical plots of instrument comparison.*

favourably with and showed slightly better results than the AU 5000 analyser. The Olympus AU 5200 analyser reaches a sustained peak performance of 300 samples/h including ISE measurements, operates reliably from day to day and is thus well suited for medium and large sized laboratories.

### References

1. Haeckel, R., Busch, E. W., Jennings, R. D., Korkholm, G. and Truchaud, A., *ECCLS Document*, **3**, 2 (1986).
2. Luley, C., Struss, H. G. and Prellwitz, W., *Journal of Automatic Chemistry*, **1** (1990).
3. Passing, H. and Bablock, W. J., *Clinical Chemistry and Biochemistry*, **22** (1984).

